# The potential use of Indian rice flour or husk in fortification of pan bread: assessing bread’s quality using sensory, physicochemical, and chemometric methods

**DOI:** 10.3389/fnut.2023.1240527

**Published:** 2023-09-15

**Authors:** Haiam O. Elkatry, Hossam S. El-Beltagi, Abdelrahman R. Ahmed, Heba I. Mohamed, Hala Hazam Al-Otaibi, Khaled M. A. Ramadan, Mohamed A. A. Mahmoud

**Affiliations:** ^1^Department of Food and Nutrition Science, College of Agricultural Science and Food, King Faisal University, Al Hofuf, Saudi Arabia; ^2^Department of Home Economics, Faculty of Specific Education, Ain Shams University, Cairo, Egypt; ^3^Department of Agricultural Biotechnology, College of Agriculture and Food Sciences, King Faisal University, Al Hofuf, Saudi Arabia; ^4^Department of Biochemistry, Faculty of Agriculture, Cairo University, Giza, Egypt; ^5^Department of Biological and Geological Sciences, Faculty of Education, Ain Shams University, Cairo, Egypt; ^6^Central Laboratories, Department of Chemistry, King Faisal University, Al Hofuf, Saudi Arabia; ^7^Department of Agricultural Biochemistry, Faculty of Agriculture, Ain Shams University, Cairo, Egypt

**Keywords:** agri-food waste, fortification, Indian red rice, phytochemicals, rheological properties, sensory attributes, chemometric analysis

## Abstract

Hassawi rice is an Indica variety cultivated in Saudi Arabia with a higher nutritional value than the commercial Basmati rice varieties. The present study has investigated the feasibility of combining Hassawi rice flour (HRF) or husk (HRHF), an abundant byproduct, with wheat flour to produce nutritious economical pan bread. To achieve this aim, the physicochemical properties of HRF and HRHF were assessed using techniques such as UPLC-tandem MS, ICP-OES, and colorimeter. The proximate composition (moisture, crude fiber, and ash) and mineral contents of HRHF are significantly (*p* < 0.05) higher than HRF. On the other hand, the compounds *p*-coumaric acid, vanillic acid, *γ*- and *δ*-tocotrienols, and *γ*-oryzanol were unique to HRF. We further determined the changes in sensory, technological, and physicochemical properties of wheat flour bread substituted with 5%, 10%, and 15% of HRF or HRHF. The rheological tests showed that the addition of HRF and HRHF increased dough development and stability time. Further, substituting wheat flour for HRF and HRHF at levels higher than 10% affected sensory attributes, such as color, taste, odor, flavor, and appearance. These changes, however, were not always at a significant level. The causes of the differences in properties between control and fortified bread samples were investigated by chemometric methods. Samples of bread + HRF at 5 and 10% had comparable overall profiles to the control. On the other hand, bread + HRHF samples proved to retain higher concentrations of bioactive molecules compared to the control bread. Our findings shed light on the possible use of rice husk fibers in baking goods, notably pan bread. Furthermore, by integrating rice husk fibers into baked goods, we may boost their health benefits while also contributing to the long-term use of agricultural waste.

## 1. Introduction

Rice is a staple crop that plays a vital role in global food security, particularly in providing sustenance for half of the world’s population (1). Hassawi rice, a reddish-brown Indica variety indigenous to Saudi Arabia’s Al-Ahsa region, offers unique nutritional benefits compared to other varieties of white rice (2). Previous studies have shown that Hassawi rice has lower levels of carbohydrates and higher amounts of protein, proximate fat, non-starch polysaccharides, and ash contents compared to white long-grain rice variants (3, 4). In addition, Hassawi rice, like other colored Indian rice varieties, contains higher levels of bioactive molecules such as phenolic compounds, flavonoids, and anthocyanins (5, 6). These natural products have been associated with various health benefits, including a reduced impact on blood glucose and insulin levels, as well as the potential to prevent chronic degenerative illnesses (5, 7–9).

Rice husk (up to 1/5 of the grain’s weight), an often-overlooked byproduct in the rice milling sector, has significant potential as a source of insoluble fibers for human consumption (10). Furthermore, rice husk contains silica, carbon compounds, and other minor minerals in substantial proportions (11). The scientific community has recognized the potential of utilizing rice husk fibers in various applications, including the development of new materials (12). The availability, affordability, biodegradability, low density, and high specific strength of rice husk products make them an attractive option for sustainable waste utilization (13–18). As a result, there is growing interest in harnessing the nutritional and functional properties of rice husk fibers to create nutritive and healthy food products that meet the demands of consumers (13–18). Technically, fibers are used in food recipes as bulking agents and fat replacers, altering consistency, texture, and sensory characteristics (13, 14, 19). Where, they can negatively impact consumer acceptance due to reduced loaf volume, increased crumb hardness, crust darkness, and might affect the taste of the final product (19–21). Despite these drawbacks, fiber supplementation offers healthy benefits thus bakery industry is continuously looking for different fiber sources that might lead to fiber-enriched breads with comparable quality to white breads (19–21). Thereby, this is one area where rice husk fibers could be utilized to produce fiber-rich bread and bakery products (22–24).

Bread and bakery products are widely consumed and have established processing lines, making them an ideal platform for incorporating rice husk fibers to improve nutritional content (22–24). Research efforts have primarily focused on using raw rice, germinated rice, and rice bran as supplements to wheat flour in bakery products (25–28). While there have been some studies exploring the incorporation of rice husk fibers, specifically from Hassawi rice, into bakery products, these efforts are relatively limited and more research is needed to fully explore the potential of using the whole grain or husk of Hassawi rice in making pan bread (22–24).

This study aims to address this research gap by examining the chemo-physical characteristics of Hassawi rice husk in comparison to whole grain flour. Furthermore, the study aims to investigate the effects of different addition ratios of Hassawi rice husk and whole grain flour to wheat flour on the rheological properties of the dough blends to determine the optimal addition ratio based on parameters such as antioxidant activity, levels of the bioactive molecules, and sensory and technological properties of the final pan bread products. These results have been validated using different chemometric methods. By conducting this research, we aim to provide valuable insights into the potential utilization of rice husk fibers in bakery products, particularly pan bread. Moreover, by incorporating rice husk fibers into bakery products, we can not only improve their nutritional content but also contribute to the sustainable utilization of agricultural waste.

## 2. Materials and methods

### 2.1. Materials

NaOH, sulfuric acid, and sodium nitrite (Merck, Germany), H_2_O_2_ 30% (ultra-pure for AA, Carlo-Erba Reagents GmbH, France), HNO_3_ 65% (ICP grade), *n*-hexane 100%, and the elements Na, K, Mg, Ca, P, Zn, Cu, Fe, Mn, Cd, Cr, Se, and Sr. standards 1,000 mg/L (ICP grade), and kuromanin (Sigma-Aldrich, United States), acetone and acetic acid (HPLC grade, CARLO ERBA Reagents GmbH, Germany), formic acid, polyvinylidenefluride, acetonitrile (HPLC grade), ethanolamine 98%, trichloroacetic acid (TCA), DPPH, ABTS, BHT, Vit C 99.9%, AlCl_3_, and catechin 96% (Sigma-Aldrich, Germany), gallic acid 98% (Fischer, United Kingdom), Folin–Ciocalteu reagent 20% (Fluka, Milwaukee, Wisconsin, United States).

Hassawi rice and husk, wheat flour (72% extraction), sucrose, corn oil, skimmed milk powder, and instant dry yeast were purchased from the local market.

### 2.2. Alkaline extraction and preparation of powdered samples

The alkaline extraction experiment was conducted to eliminate the anti-nutritional factors. To achieve this aim, 10 g of rice hulls were added to 50 mL of 2 M NaOH solution, and the mixture’s pH level was then determined. The mixture-containing screw-cap vessel was submerged in a thermostatic water bath (Water Bath, WB-2R6H-25, Bioevopeak Inc. Shandong, China) preset at 85°C. During the reaction, a stirrer worked at a speed of 200 rpm to ensure a well-mixed slurry. After 3 h, the reaction was stopped and the slurry was centrifuged. The solid residue (e.g., silica, holocellulose, and lignin) was flushed with water several times and dried before the composition analysis according to the method of Hsieh et al. (29). The samples were subsequently dried in a laboratory oven at 105°C for 3 h. Dried samples were milled for 2 min at 35,000 rpm in a laboratory mill (Huge Grinder Model No. E03407, Beijing, China) before sieving to a fine powder.

### 2.3. Flour mixes and dough preparation

Wheat flour was mixed with Hassawi rice or its husk using a spiral-bladed mixer in amounts of 5%, 10%, and 15%.

### 2.4. Rheological characteristics

The rheological properties of wheat dough blends with different ratios of Hassawi rice and its husk (5%, 10%, and 15%) were examined with the Farinograph Brabender (Brabender, Duisburg, Germany) according to the method 54-21 of AACC (30). A 300 g of flour was added to the farinograph and kneaded with a supply of water to measure the absorption of water and kneading flour. A farinogram torque measurement is used to track the dough’s resistance to mechanical stress over time. All measurements were made at room temperature (25°C) and followed the constant flour weight protocol.

### 2.5. Preparation of pan bread

Pan bread was prepared by the straight dough method according to Ahmed (31) with some modifications. To make the dough, wheat flour was combined with 1% instant dry yeast, 1% sodium chloride, 5% corn oil, 5% sugar, and 2% skimmed milk, then combined the optimum amount of water (determined by farinograph measurements of water absorption) using a Kitchen Aid Professional mixer (KPM5). Different formulas were made from various ratios of Hassawi rice and its husk (5%, 10%, and 15%, respectively). The resultant dough was divided and rounded manually, 300 g ± 5 each. The dough was fermented for 60 min at 32°C–35°C and 85% relative humidity before being baked for 15 min at 240°C–280°C in loaf pan with the dimensions 20 × 10 × 6 cm. Pan bread loaves were allowed to cool for about 1 hour before evaluation and were then packaged in polyethylene bags.

### 2.6. Physical properties

The physical characteristics of pan bread (loaf weight, volume, and specific volume) were measured according to methods 10-05.01 AACC (30). Using a balance (0.1 g), the loaf weight was measured after 2 hours at room temperature. Rapeseed displacement was used to calculate volume. Using the Collins et al. (32) approach, specific volume (cm^3^/g) was obtained by dividing loaf volume by loaf weight.

### 2.7. Determination of color

The color of the crust and crumb of bread containing Hassawi rice and its husk (5%, 10%, and 15%) was determined according to the tristimulus color system described by Francis (33) using a Minolta Colorimeter (CR 200 Japan). Color readings were expressed by Hunter values for L^*^, a^*^, and b^*^.

### 2.8. Sensory analysis

Ten trained panelists (6 males and 4 females between 33 and 55 years old) were recruited from Food Science and Nutrition Department, King Faisal University to conduct the test. They had no known illness at the time of the performance. All tested samples were made from food-grade ingredients and neither of the panelists was allergic or had intolerances to any of the ingredients used in our experiment (written consents were obtained) according to the ethical guidelines of the King Faisal University (approval number KFU-REC-2022-NOV-ETHICS819). The assessment was carried out at 25°C in a well-lit and ventilated room in two separate sessions. In each session, bread samples (labeled with a randomized code) were served in equal portions (2 cm thick slices) for the panelists, where they performed visual and ortho- and retro-nasal evaluations to assess the appearance, crust color, crumb color, crumb texture, taste, odor, and overall acceptability of samples. These attributes were selected based on previous studies (13, 34). The panelist assessed those attributes based on a 10 points numerical scale with 0.5 increments, where 0 means “strongly dislike,” 5 means “neutral,” and 10 means “strongly like.” Water was served between samples to eliminate the residual taste of previous samples.

### 2.9. Proximate composition

The following elements were measured using the AOAC method (35): moisture, protein, total fat, crude fiber, ash, total carbohydrate, and starch. The crude protein content was determined in 1 g of a dried sample using the Kjeldahl method, and moisture was measured in 5 g of a sample using the oven method (Bender ED-115, Tuttlingen, Germany) at 105°C overnight. A muffle boiler was used to estimate the sample’s ash concentration in 1 g of dried sample (Witeg FHT-14, Wertheim, Germany). After diluting, acid hydrolysis of row samples [sulfuric acid in water 2.5 (v/v %) at 80°C for 5 h], the total amount of lipids in 10 g of the acid hydrolyzed dried sample was ascertained using Soxhlet fat extraction equipment. Total resistant starch was assessed from glucose multiplied by factor 0.9 as dietary fiber, whereas total carbohydrates were measured using a spectrophotometer using a phenol-sulfuric reagent. A fully automated method was used to identify the crude fiber (FOSS-Fibertic 8000, Denmark).

### 2.10. Determination of phosphorus and potassium

The contents of the elements were determined in both Hassawi rice (HRF) and husk flour (HRHF) according to the method of AOAC (35). The potassium level was measured using a flame photometer (BWB-Flash Photometer, Berkshire, United Kingdom) and a calibration reference curve (5–25 mg/mL). Thermo Scientific Evolution 350 UV–vis spectrophotometer (Waltham, MA, United States) was used to measure phosphorus, and phosphorus concentration was computed using the standard calibration curve (*p* equivalent of 0.01–10 mg/L).

### 2.11. Determination of trace element

#### 2.11.1. Microwave digestion

According to Marin et al. (36), 2 mL of H_2_O_2_ (30%) or 8 mL of HNO_3_ (65%) were used to digest 1.0 g of the powdered sample in a closed microwave digestion/extraction (SINEO-MDS-6G SMART, China) system. Before ICP-OES analysis, the mixtures were chilled and diluted with 25 mL of ultrapure water from the Milli-Q system (Millipore, France).

#### 2.11.2. Trace element levels determination by ICP-OES

An autosampler of AS 93-plus Argon (purity >99.995%) was used to maintain plasma and carrier gas. Trace element concentrations were measured using an ASX-280 Autosampler-equipped Shimadzu ICP-OES (9820 Series, Tokyo, Japan). Calibration was achieved using a multi-element standard solution and the calibration range for each element’s calibration range ranged from 0 to 10 mg/L.

### 2.12. Extraction of the total phenolic fractions

A lipophilic fraction of the whole grain rice sample and rice husk was obtained by extracting each sample (0.5 g) twice with 5 mL of 100% *n*-hexane at 45°C for 3 h in screw cap tubs for removing the lipid fraction which interfaces with phenolic determination. After extraction, the lipid fraction was discarded, and the solid residue was dried using rotary evaporator (335 mbar, 55°C). A free phenolic fraction was obtained by extracting the total phenolics from lipophilic free residual using 5 mL of acetone/water/acetic acid (70/29.5/0.5, v/v/v) for 2 h at 25°C with shaking (Witeg shaking water bath, Wertheim, Germany). The supernatant was collected after centrifugation at 10000 × g for 20 min at 20°C (HERMEL-Z36HK—Germany), and the extraction was carried out once more. Each supernatant was combined. The extract was first reconstituted in 10 mL of 100% acetonitrile (due to the complete dissolving of the extract residues) after being evaporated under low pressure, dried, and weighted. This reconstituted extract was used to determine total phenolic, flavonoids, antioxidant activity and UPLC-MSMS separation of phenolic compounds.

### 2.13. Determination of total phenolic concentrations

With a few modifications, the total phenolic content was calculated following Goffman and Bergman (37). A mixture of 0.1 mL of the sample extract, 0.50 mL of deionized water, 0.25 mL; of 20% Folin–Ciocalteu reagent, and 0.5 mL of 0.5 M ethanolamine was added after 5 min. The absorbance of the combination was measured at 760 nm after 90 min at room temperature using a Thermo Scientific Evolution 350 UV–vis spectrophotometer (MA, United States). Total phenolic content was defined as 100 mg gallic acid (GA)/100 g of sample (DW) based on the standard curve of gallic acid with concentration ranged from 0–400 μ/mL ( $Y=0.019X+0.003;R^{2}=0.99$ ).

### 2.14. Determination of total flavonoid concentrations

The total flavonoid content was determined using the spectrophotometric approach reported by Chang et al. (38). 0.25 mL of the extract was added to 1 mL of deionized water and 0.075 mL of 5% sodium nitrite (w/v). After 5 min, 0.15 mL of 10% AlCl_3_ (w/v) was added to the mixture. After 6 min, 0.5 mL of 1 M NaOH was added. Next, 0.5 mL of deionized water was added. After centrifugation at 8000 g for 4 min at room temperature (HERMEL-Z36HK—Germany), the optical density was measured at 510 nm using a Thermo Scientific Evolution 350 UV–vis spectrophotometer (MA, United States) against the reagent blank. Catechin (CE)/100 g sample was used to represent the total flavonoid content (DW). The total flavonoids content were calculated based on a plotted standard curve of catechin with concentrations of 5–200 mg/mL ( $Y=0.977X-0.005;R^{2}=0.99$ ).

### 2.15. Determination of total anthocyanin concentrations

Total anthocyanins were extracted, and their concentrations were calculated using the Abdel-Aal et al. method (39). Samples (0.5 g) were extracted in 5 mL of acidified methanol (85% methanol and 15% 1.5 N HCl, v/v) for 40 min at room temperature. After centrifugation at 20000 g for 20 min at 4°C (HERMEL-Z36HK—Germany), the supernatant was transferred to a 10 mL volumetric flask, and the extraction was then repeated in the supernatant. Supernatants were mixed. At 535 nm, the mixture’s absorbance was measured against a blank for the reagent. The total anthocyanin concentration was calculated using the average molar extinction coefficient of kuromanin and represented as mg kuromanin (KE)/100 g sample (DW). The concentration range (2.5–20.0 mg of KE) was used to create the calibration standard curve for kuromanin, and the average molar extinction coefficient (*Ɛ*) was 25,700 ± 110 M^−1^ cm^−1^.

### 2.16. Determination of DPPH radical scavenging capacities

To measure the 2,2-diphenyl-1-picrylhydrazyl (DPPH) radical scavenging capacity, Goffman and Bergman method (37) was modified. The sample extract was combined with 0.9 mL of DPPH solution (80 mg/L in 100% methanol) and incubated at room temperature for 30 min while kept in the dark, and its absorbance was then read at 517 nm. Using the following formula, the percentages of DPPH radical scavenging activities in samples were calculated:

$$\mathrm{DPPH activity}\;\left(\%\right)=\frac{A_{0}-A_{1}}{A_{0}}\times 100$$

where *A*_0_ is the absorbance of the blank and *A*_1_ is the absorbance of the sample.

### 2.17. ABTS radical scavenging assay

The assay measures a substance’s capacity to scavenge a radical cation of 2,2′-azino-bis ethylbenzthiazoline-6-sulfonic acid (ABTS) in relation to a reference (BHT) according to the method of Re et al. (40). The radical cation was made by combining 2.45 mM potassium persulfate (1/1, v/v) with 7 mM ABTS stock solution over a period of 4 to 16 h, depending on how quickly the reaction was finished and the absorbance stabilized. For measurements, ethanol was used to dilute the ABTS solution. After mixing 0.9 mL of ABTS^+^ with 0.1 mL of the test samples for 45 s, the photometric assay was performed, and measurements were made at 734 nm after 1 min. The reduction in absorbance at various doses was used to calculate the antioxidant activity of the tested samples and standards (BHT and vitamin C) by using the following equation:

$$E=\frac{A_{c}-A_{t}}{A_{c}}\times 100$$

where: *A*_t_ and *A*_c_ are respective absorbance of tested samples or standard and ABTS^+^.

### 2.18. UPLC-MS/MS identification of total phenolic compounds

Identification of the free phenolic compounds was achieved using Waters Acquity UPLC–I class coupled with Xevo TQD MS (United States), Acquity UPLC BEH C18 1.7 μm–2.1 × 100 mm column flow rate 0.5 mL min^−1^, the injection volume 5 μL, Masslynix v4.1 software with Mass library, argon as collision cell gas inlet 7 psi, nitrogen pressure 60 psi. The MS was set to an atmospheric pressure electrospray ionization (ESI) source, operated in negative ion mode. The electrospray capillary voltage was set to 3,000 V, with a nebulizing gas flow rate of 12 L/h and a drying gas temperature of 300°C. Mass spectrometry data were acquired in the Scan mode (mass range *m*/*z* 100–1,000). To scan the total phenolic profile, a binary gradient of (A) 0.5% formic acid in deionized water and (B) 100% methanol at 0.8 mL/min at 25°C. The gradient used was: 0 min, 15% B; 0 to 15 min, linear gradient to 15% B; 15 to 25 min, linear gradient to 25% B; 25 to 35 min, linear gradient to 50% B; 35 to 50 min, linear gradient to 75% B; 50 to 55 min, linear return to 15% B; and 55 to 57 min, isocratic at 100% B to re-equilibrate. An internal calibration standard was used for quantification. Mass spectral data were compared by the mass metabolite spectral library. The metabolite library uses the high mass accuracy MS/MS spectra, Rt, and isotopic information to identify and confirm compounds.

### 2.19. Identification of tocopherols, tocotrienols, and *γ*-oryzanol

Rice grain fine powder and rice husk powder were extracted with 100% methanol using a sample-to-solvent ratio of 1:33 (w/v). The mixture was flushed with nitrogen gas and shaken overnight at room temperature. After centrifugation at 2000 × g for 10 min at room temperature (HERMEL-Z36HK—Germany), the supernatant was filtered through a 0.45 μm polyvinylidenefluoride (PVDF) membrane. Waters Acquity UPLC-I class coupled with Xevo TQD MS (United States), Acquity UPLC BEH C18 1.7 μm–2.1 × 100 mm column flow rate 0.8 mL min^−1^, the injection volume 20 μL. The filtrate was eluted with a gradient mobile phase consisting of (A) 100% acetonitrile, (B) 100% methanol, and (C) 1% acetic acid in 50% methanol at 0.5 mL/min at 25°C controlled by an HPLC column heater (TL-105, Timberline Instruments, Boulder, Colo., United States). The gradient was used as follows: 0–1 min, 45% A, 35% B, and 20% C; 1–2 min, linear gradient to 45% A, 45% B, and 10% C; 2–16 min, linear gradient to 30% A, 65% B, and 5% C; 16–20 min, linear gradient to 25% A and 75% B; 20–22 min, linear gradient to 100% B; 22–25.4 min, isocratic at 100% B; 25.4–25.5 min, linear return to 45% A, 35% B, and 20% C; 25.5–35 min, isocratic at 45% A, 35% B, and 20% C to re-equilibrate. The peak identification of tocopherols, tocotrienols, and *γ*-oryzanols was performed by comparing their mass spectra with those of standards in the metabolite library. The quantification of tocopherols, tocotrienols, and oryzanol was performed by an external calibration curve.

### 2.20. Statistical analysis

Data treatment and preparation for statistical analysis followed the report of Mahmoud et al. (41). First, the dataset was tested for outliers using the Gruber test and then normalized using the *z*-score. Sensory results were averaged and plotted on a radar chart with a 10 points scale. The analysis of variance for all treatments was performed using SPSS version 16. Based on the number of samples, either student *t*-test or Duncan’s preference test was employed to distinguish between the treatments. The mean ± standard deviation of three replicates was used. Hierarchical clustering based on heatmap analysis of samples, followed by a nearest neighbors Spearman correlation ranking test to identify the treatment that is closest to the control using Morpheus Open.^1^ The principal component analysis is used to group variables based on their correlation with samples using XLSTAT 2022^®^ (Addinsoft, Paris, France).

## 3. Results and discussion

### 3.1. Characterization of rice raw materials

#### 3.1.1. Proximate analysis

The proximate chemical analysis of HRHF and HRF is reported in Table 1. Generally, HRF contained uniquely fat (0.69%), protein (8.29%), total nitrogen (1.33%), carbohydrate (74.50%), and total resistant starch (1.03%). Similar proximate composition were found in pigmented rice varieties as reported by Petroni et al. (42) and Reddy et al. (43). On the other hand, HRHF have higher concentrations of moisture, ash, and crude fiber contents as compared with HRF. Similar results on ash content were reported by Melini et al. (44). Additionally, having higher moisture content is attributed to the water binding capacity of food fibers (45). Though, the reduction in proximate contents in HRHF compared to HRF is attributed to the hulling and milling process of rice paddies (43).

**Table 1:** Proximate analysis of HRF and HRHF.

Parameters	HRF	HRHF
Moisture %	10.89 ± 0.47^b^	13.38 ± 0.11^a^
Fat %	0.98 ± 0.01	0.0
Protein %	8.29 ± 0.32	0.0
Total nitrogen %	1.33 ± 0.05	0.0
Ash %	0.69 ± 0.01^b^	19.90 ± 0.20^a^
Carbohydrate %	74.50 ± 1.11	0.0
Crude fiber %	14.33 ± 0.27^b^	78.30 ± 0.98^a^
Total resistant starch %	1.03 ± 0.05	0.0

Each data is average ± standard deviation across three distinct replications. According to student’s two-sample *t*-test, the letters in the same column display a significant difference at *p* ≤ 0.05. ^*^Protein % calculated from the total % of nitrogen × 6.25.

#### 3.1.2. Mineral contents

Generally, HRHF contained higher concentrations of elements compared with HRF (Table 2). This is specifically true for silica levels as reported by Alshatwi et al. (46). The higher elemental concentration in HRHF might be attributed to the higher ash and fiber contents. Previous reports have indicated the positive correlation between the ash % and many elements withing the same matrix (46–48).

**Table 2:** Minerals content (mg/100 g DW) of HRF and HRHF.

Sample	HRF	HRHF
K	19.70 ± 0.20^b^	50.4 ± 0.23^a^
Mg	4.92 ± 0.12^b^	20.03 ± 0.19^a^
Ca	4.00 ± 0.09^b^	13.29 ± 0.05^a^
Na	2.41 ± 0.20^b^	8.02 ± 0.52^a^
Fe	0.82 ± 0.02^b^	5.34 ± 0.34^a^
Zn	0.084 ± 0.001^b^	4.31 ± 0.05^a^
Cu	0.062 ± 0.001^b^	0.89 ± 0.004^a^
P	2.61 ± 0.04^b^	11.21 ± 0.11^a^
Si	20.84 ± 1.002^b^	92.33 ± 4.60^a^
Al	0.003 ± 0.00^b^	0.78 ± 0.02^a^

Each data is the average ± standard deviation across three distinct replications. According to student’s two-sample *t*-test, the letters in the same row display a significant difference at *p* ≤ 0.05.

#### 3.1.3. Antioxidants activity

The antioxidant activity of HRF and HRHF is presented in Table 3. The data demonstrate that the DPPH % and ABTS % scavenging activities considerably enhanced as HRF and HRHF concentrations were raised from 50 to 400 μg/mL. The higher concentrations of DPPH (96.06% and 96.13%) and ABTS (96.04% and 97.01%) were detected in 400 μg/mL HRF and HRHF, respectively, in comparison to 400 μg/mL Vit C and BHT, which were used as standards. The IC_50_ of DPPH (2.1 and 2.8 μg/mL) and ABTS (2.5 and 3.1 μg/mL) was detected in HRF and HRHF, respectively. Antioxidant activity is measured by the effective concentration at 50% ROS scavenging (IC_50_); the lower the IC5_0_, the higher the antioxidant activity of the extracts. The HRF and HRHF extracts show significant antioxidant activity, as demonstrated by a comparison to the IC_50_ values of the conventional vitamin C and BHT.

**Table 3:** Antioxidant activity of HRF and HRHF.

Samples	% inhibition of DPPH					% inhibition of ABTS				
	Concentration (μg/mL)					Concentration (μg/mL)				
	50	100	200	400	IC_50_	50	100	200	400	IC_50_
HRF	24.07 ± 1.78^b^	38.96 ± 1.06^b^	72.08 ± 1.02^b^	96.06 ± 1.12^a^	2.1	22.3 ± 0.15^b^	36.33 ± 0.24^b^	71.44 ± 0.46^a^	96.04 ± 2.84^a^	2.5
HRHF	28.92 ± 0.34^a^	44.63 ± 0.52^a^	74.83 ± 0.87^a^	96.13 ± 0.82^a^	2.8	25.8 ± 0.29^a^	40.95 ± 1.1^a^	73.43 ± 3.58^a^	97.01 ± 1.42^a^	3.1
Vit C	20.43 ± 0.25^c^	34.98 ± 0.42^d^	71.55 ± 0.87^b^	95.36 ± 1.16^bc^	12.9	19.39 ± 0.61^d^	33.36 ± 0.71^c^	66.15 ± 0.37^c^	91.66 ± 1.55^c^	14.35
BHT	21.2 ± 0.61^c^	37.09 ± 1.07^c^	69.65 ± 2.21^c^	93.58 ± 2.26^c^	11.2	20.31 ± 0.2^c^	36.73 ± 0.54^b^	69.73 ± 0.83^b^	92.27 ± 1.14^b^	12.08

Each data is the average ± standard deviation across three distinct replications. According to Duncan’s test, the letters in the same column display a significant difference at *p* ≤ 0.05.

#### 3.1.4. Secondary metabolites

**Figure 1:**
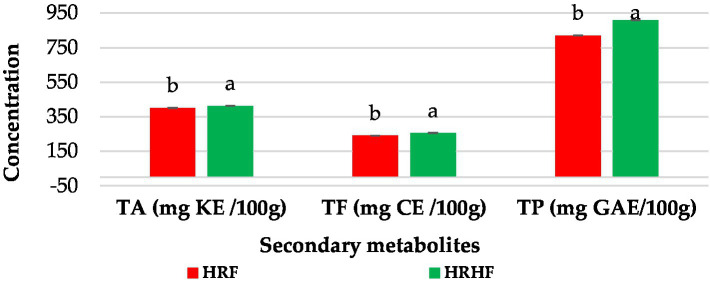
Secondary metabolites of rice raw material (TA: total anthocyanins, TF: total flavonoids, and TP: total phenols). Each data is the average ± standard deviation across three distinct replications. According to student’s two-sample *t*-test, the letters display significant differences at *p* ≤ 0.05.

Total phenolics (TP), total flavonoids (TF), and total anthocyanin (TA) contents of HRF, HRHF, and bread are presented in Figure 1. The analysis of secondary metabolites showed that HRHF contained higher concentrations of TP (909.14 and 820.72 mg GAE/100 g), TF (256.04 and 240.73 mg CE/100 g), and TA (414.02 and 400.97 mg KE/100 g) than HRF. The composition of these fractions has been explored using UPLC-MS/MS. Thereby, the ethanolic extracts of HRF and HRHF proved to contain 11 and 8 phenolic compounds when analyzed by UPLC-MS/MS (Table 4 and Supplementary Figures S1, S2). The most abundant constituents were 3-hydroxycinnamic acid (343.59 ± 32.7 mg100 g^−1^ HRF and 283.83 ± 27.29 mg 100 g^−1^ HRHF), epigallocatechin (81.56 ± 7.76 mg 100 g^−1^ HRF and 88.19 ± 8.47 mg 100 g^−1^ HRHF), and ferulic acid (79.25 ± 7.54 mg 100 g^−1^ HRF and 103.37 ± 9.93 mg 100 g^−1^ HRHF).

**Table 4:** LC-tandem MS identification of phenolic compounds content in HRF and HRHF extracts.

	Compound name	Ret. time (min)	(M + H)^−^	Base beak *m*/*z*	mg 100 g^−1^ DW		Antioxidant activity
					HRF	HRHF	
1	Gallic acid	2.210	169	125	21.40 ± 2.03	0.0	Strong (49)
2	Protocatechuic acid	23.13	153	109	32.39 ± 3.08^b^	46.72 ± 4.49^a^	Highly active (50)
3	Caffeic acid	23.74	179	135	9.26 ± 0.88^b^	12.26 ± 1.17^a^	Active (51)
4	Chlorogenic acid	25.65	353	191	24.87 ± 2.36^a^	12.26 ± 1.17^b^	
5	Vanillic acid	27.42	167	152	9.26 ± 0.88	0.0	
6	Vanillin	28.21	151	136	8.68 ± 0.82^b^	12.26 ± 1.17^a^	
7	*p*-Coumaric acid	31.50	163	119	4.63 ± 0.44	0.0	Active (52)
8	Ferulic acid	33.01	193	178	79.25 ± 1.54^b^	103.37 ± 2.93^a^	Active (53)
9	3-Hydroxycinnamic acid	33.28	163	119	343.59 ± 3.7^a^	283.83 ± 2.29^b^	
10	Epigallocatechin	33.81	305	163	81.56 ± 1.76^b^	88.19 ± 1.47^a^	Strong (53)
11	Rutin	35.60	609	609	4.05 ± 0.38^a^	2.92 ± 0.28^b^	

Each data is the average ± standard deviation across three distinct replications. According to student’s two-sample *t*-test, the letters in the same row display a significant difference at *p* ≤ 0.05.

Adom and Liu (54) showed that corn, wheat, oats, and rice had more than 93% of ferulic acids in the bound form, which was consistent with the present study’s findings. Further, gallic, vanillic, and *p*-coumaric acids were unique to HRF. From these three, vanillic and *p*-coumaric acids were previously detected in red and black rice grains (55).

Seven and four tocopherols were identified in their extracts using LC-tandem-MS (Table 5 and Supplementary Figure S3). The compounds *γ*- and *δ*-tocotrienol were the main constituents in HRF, while they were not identified in HRHF. The major common tocopherol among them was α-tocopherol (36.29 ± 0.33 mg kg^−1^ in HRF and 39.72695 ± 0.17 mg kg^−1^ in HRHF). The other unique compound to HRF was *γ*-oryzanol (246.99 ± 1.23 mg kg^−1^). All the identified tocopherols were previously reported in various rice variants either raw whole rice, rice bran, or germinated rice bran (56, 58, 59). Among them, *γ*-oryzanol is unique to rice varieties in general (58). There are reports linked between the content of all types of tocopherols (*α*, *β*, and *γ*) and oryzanol and protection from cardiovascular diseases and lower plasma cholesterol which is considered a major nutritional benefit of fortification with rice or husk (56, 60). Further, they possess antioxidant activity that can protect against ROS (56, 60).

**Table 5:** LC-tandem-MS identification of tocopherols, tocotrienols, and *γ*-oryzanol compounds content in HRF and HRHF extracts.

	Compound name	Ret. time (min)	(M + H)^−^	Base beak *m*/*z*	mg 100 g^−1^ DW		Antioxidant activity
					HRF	HRHF	
1	*α*-Tocopherol	0.49	429.38	237	3.63 ± 0.33^a^	3.97 ± 0.17^a^	Strong (56, 57)
2	*α*-Tocotrienol	1.52	423.33	423	1.76 ± 0.16^a^	0.94 ± 0.04^b^	Highly strong (56, 57)
3	*γ*-Tocopherol	3.71	415.36	239	2.11 ± 0.19^a^	1.54 ± 0.07^b^	Active (56, 57)
4	*γ*-Tocotrienol	4.95	395.30	195	6.97 ± 0.63	0.0	Active (56, 57)
5	*δ*-Tocopherol	5.82	401.34	179	3.24 ± 0.29^a^	1.16 ± 0.05^b^	Active (56, 57)
6	*δ*-Tocotrienol	6.42	409.30	199	5.52 ± 0.50	0.0	Active (56, 57)
7	*γ*-Oryzanol	7.39	601.90	601.90	24.70 ± 1.23	0.0	Strong (56, 57)

Each data is the average ± standard deviation across three distinct replications. According to student’s two-sample *t*-test, the letters in the same row display a significant difference at *p* ≤ 0.05.

#### 3.1.5. Color

**Figure 2:**
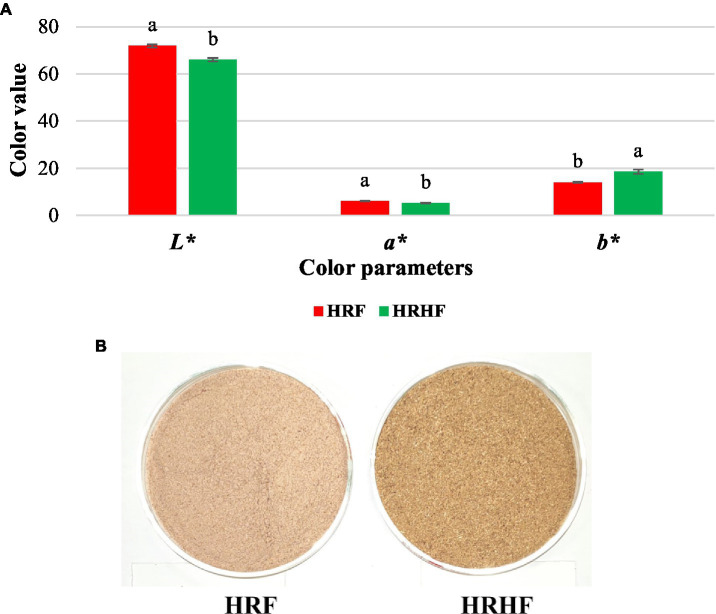
Color values of raw materials **(A)** with illustration of the actual samples **(B)**. Each data is the average ± standard deviation across three distinct replications. According to student’s two-sample *t*-test, the letters display significant differences at *p* ≤ 0.05.

Color measurements of samples indicated that husk flour was significantly lower in brightness (L^*^; *p* = 0.001) and redness (a^*^; *p* = 0.002) while having higher yellowness (b^*^; *p* = 0.001) compared to the whole-grain rice flour (Figure 2). This might be attributed to the concentration of pigments, mostly anthocyanins, in the outer layers compared to the endosperm (61). Similarly, the TA levels in HRHF were significantly higher compared to HRF samples as reported in the previous section. Our finding is in-line with the results of Reddy et al. (43) who reported that rice polishing increase the lightness of rice samples in response to the decrease in the total anthocyanins contents.

To conclude for this part, the chemical and physical variables of HRF and HRHF indicated that the whole grain flour was generally higher in proximate components and plant tocopherols, tocotrienols, and *γ*-oryzanol as well as the color parameters. It is known that the macromolecules fat, protein, and carbohydrates are concerted in the grain rather than the husk. Further, tocopherols, tocotrienols, and *γ*-oryzanol are fat-soluble molecules concentrated in the germ and the bran (57). On the other hand, the husk flour showed higher phytochemical and elemental contents and higher antioxidant activities. Previous reports indicated that rice husk is rich in polyphenols, anthocyanins, and flavonoids which protect the seeds against oxidative stress (62). However, the results of the LC-tandem-MS indicated that the whole rice grain has a richer profile compared to the husk. We presume that it is attributed to the condensed polyphenols concentrated in the husk layers, and these compounds needed different extraction methods and a higher mass range than the ones used for our experiment (62, 63).

### 3.2. Dough mixing properties

Dough blends of control bread and bread prepared by the addition of 5%, 10%, and 15% of HRF or HRHF were prepared, and their properties were examined using farinograph analysis. The results showed that the bread samples fortified with HRF or HRHF exhibited significant changes in water absorption %, dough stability, mixing tolerance index (except for bread + HRF 10%), farinograph quality number, and the time for the dough to breakdown. As for the dough development time, only three samples were significantly different from the control. These were bread + HRF at 5% and bread + HRHF at 5% and 15% (Table 6).

**Table 6:** Farinograph parameters of bread substituted with different levels of HRF and HRHF.

Samples		Water absorption (WA) %	Dough development time (DTT) min	Dough stability min	Mixing tolerance index (MTI) BUE*	Farinograph quality number	Time to breakdown
Bread control		62.03 ± 0.06^a^	1.29 ± 0.03^c^	2.33 ± 0.10^e^	48.67 ± 3.06^c^	30.33 ± 1.53^g^	2.66 ± 0.10^f^
Bread + HRF %	5	60.00 ± 0.50^b^	1.35 ± 0.03^c^	3.33 ± 0.08^d^	56.33 ± 3.06^a^	36.33 ± 1.53^f^	3.35 ± 0.08^e^
	10	58.60 ± 0.10^c^	1.48 ± 0.07^b^	3.53 ± 0.09^c^	49.00 ± 4.00^c^	39.00 ± 1.00^e^	3.5 ± 0.03^d^
	15	57.40 ± 0.10^d^	1.54 ± 0.09^b^	4.22 ± 0.04^b^	40.33 ± 1.53^e^	45.00 ± 1.00^b^	4.35 ± 0.03^c^
Bread + HRHF %	5	59.37 ± 0.21^b^	1.35 ± 0.03^c^	3.33 ± 0.03^d^	45.00 ± 2.00^d^	38.33 ± 1.53^d^	3.45 ± 0.03^d^
	10	58.50 ± 0.10^c^	1.32 ± 0.02^c^	10.32 ± 0.11^a^	35.67 ± 2.52^f^	43.00 ± 3.61^c^	4.45 ± 0.07^b^
	15	57.93 ± 0.15^d^	1.89 ± 0.59^a^	10.48 ± 0.03^a^	53.67 ± 2.08^b^	122.67 ± 1.53^a^	12.16 ± 0.09^a^

Each data is the average ± standard deviation across three distinct replications. According to Duncan’s test, the several letters in the same column display a significant difference at *p* ≤ 0.05.

The quantity of water used to reach the desired consistency is known as “water absorption.” It speaks to the capacity of flour to bind the most water with the least amount of further mixing required for the creation of dough (64). Table 6 displays the mixing characteristics of dough formulations after the addition of HRF and HRHF at various levels. The control dough has a high capacity for absorbing water (62.03%). As the HRF and HRHF ratios in the dough increased, the water absorption capacity significantly decreased. The formation of the gluten network, and therefore the quality of the bread, is significantly influenced by water absorption (65).

The observed variation in water absorption capacity may be explained by using rice bran in place of gluten-containing wheat flour, which reduced water-fiber interaction and increased the hydrophobicity of the dough (66). Additionally, the fiber in HRF and HRHF reduces the dough’s ability to absorb water and its tolerance for mixing as well as its tenacity and extensibility (65).

Dough development time (min) is the amount of time between adding water and reaching the dough’s maximum consistency before the process of weakening begins (67). In comparison to control bread, the development time decreased as HRF and HRHF levels were increased. The dough development time is intimately related to the gluten characteristics of wheat flour because gliadins and glutenin provide the dough with its traits of elasticity and robustness and allow the building of a protein network that other forms of flour do not exhibit (66, 67). The results are in close agreement with those made by other authors after adding HRF and HRHF. The results show that the addition of HRF and HRHF disrupts the starch-protein matrix, which can reduce dough elasticity and weaken the dough during mixing (68) and other gluten-free ingredients, such as pea flour (64).

The disparity between arrival and departure time is known as “dough stability” and shows dough strength (65). Additionally, in comparison to bread control, the dough stability, farinograph quality number, and the time for the dough to breakdown were significantly increased with increasing concentrations of HRF and HRHF (Supplementary Figure S4).

### 3.3. Chemical and physical characteristics of the final product

#### 3.3.1. Proximate analysis

**Figure 3:**
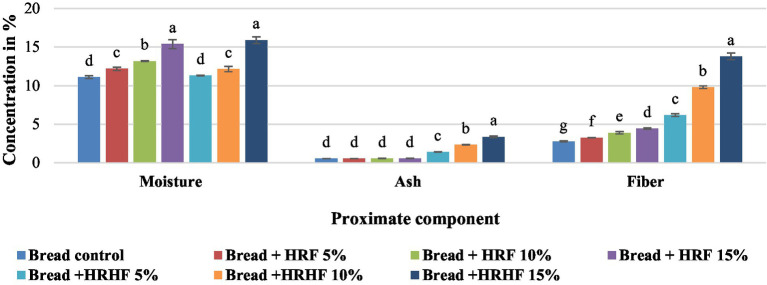
Proximate analysis of pan bread substituted with HRF and HRHF at different levels. Each data is the average ± standard deviation across three distinct replications. According to Duncan’s test, the letters display significant differences at *p* ≤ 0.05.

The proximate composition of the bakery products (control and supplemented bread samples) is presented in Figure 3. The data show that the percentage of moisture, ash, and fiber content was significantly higher in bread substituted with all levels of HRHF and HRF as compared to control bread. However, the most pronounced increase in moisture (30.9 and 26.8%), ash (522 and 20.4%), and fiber (396 and 59%) was detected in bread substituted with 15% HRHF and HRF, respectively.

Our results are in line with the findings of De Delahaye et al. (69) who studied the effects of adding rice bran flour containing higher dietary fiber content to pizza dough recipes on the moisture, fiber, and ash contents and the overall acceptability of the final products. The higher elemental levels are correlated with the higher ash contents as reported by Melini et al. (44) and da Rocha Lemos Mendes et al. (70).

#### 3.3.2. Mineral contents

Macro, micro, and trace elements bakery products are reported in Table 7. Also, compared to the control bread, the bread produced with various amounts of HRF and HRHF (5%, 10%, and 15%) had significantly higher mineral contents. Our findings could be supported by previous results showing that the use of bakery products made from or supplemented with rice flour contributed to the increase in Zn, Fe, K, Mg, and P levels in the final products (71–73).

**Table 7:** Minerals contents (mg/100 g DW) of Bread substituted with different levels of HRF and HRHF.

Treatments		K	Mg	Ca	Na	Fe	Zn	Cu	P	Se	Mn	Si	Al
Bread control		0.38 ± 0.01^g^	0.04 ± 0.01^g^	13.70 ± 0.05^b^	0.15 ± 0^e^	3.78 ± 0.22^d^	0.87 ± 0.05^d^	0.0	0.15 ± 0^e^	0.09 ± 0^a^	0.6 ± 0^a^	0.0	0.0
Bread + HRF %	5	1.38 ± 0.08^f^	0.29 ± 0.02^f^	15.06 ± 0.25^a^	0.26 ± 0^d^	3.96 ± 0.07^d^	0.93 ± 0.02^d^	0.0	0.18 ± 0.01^c^	0.09 ± 0^a^	0.55 ± 0.02^b^	0.0	0.0
	10	2.15 ± 0.1^e^	0.49 ± 0.02^e^	14.31 ± 0.08^a^	0.39 ± 0.02^c^	3.89 ± 0.16^d^	0.90 ± 0.04^d^	0.0	0.16 ± 0^d^	0.08 ± 0^b^	0.51 ± 0.03^c^	0.0	0.0
	15	3.00 ± 0.16^d^	0.70 ± 0.04^d^	15.38 ± 0.82^a^	0.51 ± 0.03^b^	4.06 ± 0.05^bc^	0.96 ± 0.04^d^	0.0	0.18 ± 0^c^	0.08 ± 0^b^	0.52 ± 0.01^c^	0.0	0.0
Bread + HRHF %	5	3.21 ± 0.08^c^	1.17 ± 0.03^c^	14.66 ± 0.68^a^	0.40 ± 0.01^c^	4.25 ± 0.1^ab^	1.21 ± 0.06^c^	0.05 ± 0^c^	0.66 ± 0.05^c^	0.08 ± 0.01^b^	0.53 ± 0.04^c^	13.8 ± 0.33^c^	0.03 ± 0^b^
	10	5.33 ± 0.30^b^	2.24 ± 0.10^b^	14.44 ± 0.8^a^	0.49 ± 0.01^b^	4.03 ± 0.18^c^	1.32 ± 0^b^	0.09 ± 0.01^b^	1.30 ± 0.05^b^	0.08 ± 0^b^	0.56 ± 0.02^b^	59.69 ± 0.22^b^	0.08 ± 0^b^
	15	8.40 ± 0.03^a^	3.25 ± 0.01^a^	15.24 ± 0.08^a^	0.54 ± 0.07^a^	4.32 ± 0.03^a^	1.44 ± 0.07^a^	0.14 ± 0.01^a^	1.87 ± 0.11^a^	0.08 ± 0^b^	0.48 ± 0.03^d^	104.23 ± 1.46^a^	0.12 ± 0.01^a^

Each data is the average ± standard deviation across three distinct replications. According to Duncan’s test, the letters in the same column display a significant difference at *p* ≤ 0.05.

#### 3.3.3. Bioactive molecules and antioxidants activity

Table 8 shows the antioxidant activity of the control bread and the bread made with additions of 5%, 10%, and 15% HRF and HRHF. According to the research, bread substituted with 5%, 10%, and 15% of HRF and HRHF greatly boosted its ability to scavenge DPPH and ABTS when its concentration was increased from 25 to 200 μg/mL. At 200 μg/mL extract, higher concentrations of DPPH (51.70% and 57.70%) and ABTS (50.18% and 54.48%) were detected in bread prepared with 15% HRF and HRHF, respectively, when compared to bread control. Similar results were reported by Mau et al. (74), who observed that the chiffon cakes’ antioxidant activity was significantly increased by the addition of black rice.

**Table 8:** Antioxidant activity of bread containing substituted flour with different levels of HRF and HRHF.

Samples		% Inhibition of DPPH				% Inhibition of ABTS			
		Concentration (mg/mL)				Concentration (mg/mL)			
		25	50	100	200	25	50	100	200
Bread control		2.47 ± 0.05^j^	3.66 ± 0.42^h^	4.88 ± 0.15^i^	6.9 ± 0.1^g^	1.79 ± 0.04^h^	3.86 ± 0.09^h^	4.03 ± 0.1^g^	5.43 ± 0.13^f^
IC_50_			1250.8			1924.47			
Bread + HRF %	5	4.77 ± 0.22^h^	15.41 ± 0.27^f^	25.46 ± 0.12^h^	48.83 ± 0.78^e^	2.89 ± 0.14^g^	9.47 ± 0.19^g^	20.23 ± 0.51^f^	38.51 ± 0.89^e^
	10	5.39 ± 0.17^g^	18.7 ± 0.06^e^	27.54 ± 0.16^g^	46.92 ± 0.08^f^	5.64 ± 0.22^e^	16.5 ± 0.15^e^	27.32 ± 0.4^d^	46.28 ± 0.28^d^
	15	9.56 ± 0.24^f^	19.83 ± 0.65^f^	31.59 ± 0.23^d^	51.71 ± 0.4^e^	7.09 ± 0.22^f^	18.85 ± 0.21^d^	30.31 ± 0.51^c^	50.18 ± 0.53^e^
IC_50_ Bread + HRF %	5	201.9				221.1			
	10	194.9				194.3			
	15	167.8				177.4			
Bread + HRHF %	5	6.38 ± 0.13^f^	14.66 ± 0.19^g^	28.98 ± 0.09^f^	48.3 ± 0.32^e^	4.32 ± 0.07^f^	12.71 ± 0.19^f^	21.66 ± 0.33^e^	45.38 ± 0.69^d^
	10	10.55 ± 0.25^d^	19.08 ± 0.17^d^	29.6 ± 0.49^e^	50.15 ± 0.17^d^	7.36 ± 0.11^d^	18.14 ± 0.26^d^	27.97 ± 0.18^d^	50.34 ± 2.32^c^
	15	12.38 ± 0.42^c^	21.19 ± 0.27^e^	33.07 ± 0.26^d^	57.70 ± 0.11^b^	11.12 ± 0.17^c^	19.53 ± 0.95^d^	30.81 ± 0.58^d^	54.84 ± 0.16^b^
IC_50_ Bread + HRHF %	5	197.5				256.2			
	10	202.3				207.6			
	15	182.5				189.8			

Each data is the average ± standard deviation across three distinct replications. According to Duncan’s test, the letters in the same column display a significant difference at *p* ≤ 0.0.

**Figure 4:**
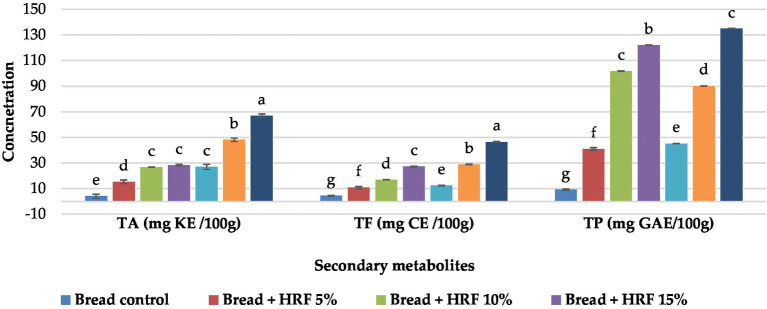
Secondary metabolites of bakery products (TA: total anthocyanins, TF: total flavonoids, and TP: total phenols). Each data is the average ± standard deviation across three distinct replications. According to student’s two-sample *t*-test, the letters display significant differences at *p* ≤ 0.05.

The results revealed that fortification of wheat flour with either HRF or HRHF has led to significantly increasing the contents of TP, TF, and TA compared to control bread proportionally to the addition level (5%, 10%, and 15%; Figure 4). Our findings are comparable to those of Mau et al. (74) who reported that increasing the amount of black rice powder caused a considerable rise in the concentrations of total phenols and total anthocyanins in chiffon cakes.

Our study found that, apart from the TP content at a 10% addition, the levels of TP, TF, and TA were higher in bread supplemented with HRHF. However, these contents were smaller than the amounts reported in the raw HRF and HRHF. This could be attributed to the thermal treatment during the baking process that initiates a series of polymerization and decarboxylation of phenolic compounds, ultimately leading to a decrease in the original levels existing in the raw materials acids (75). The same is true for the other natural constituents, TF, and TA (75, 76).

#### 3.3.4. Physical characterization of bread products

**Figure 5:**
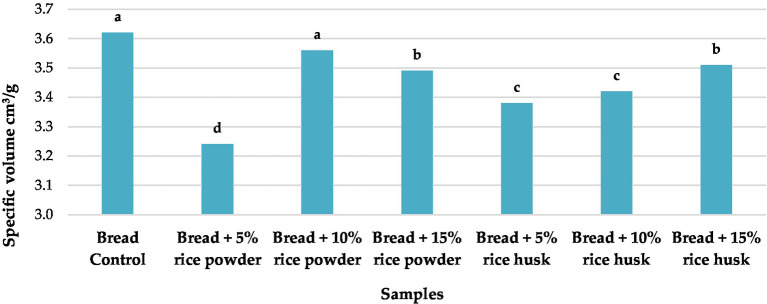
Specific volume of pan bread substituted with HRF and HRHF at different levels. Each data is the average ± standard deviation across three distinct replications. According to Duncan’s test, the letters display significant differences at *p* ≤ 0.05.

The physical characterization of these products showed that the addition of different concentrations of HRF and HRHF caused significant changes in weight, height, and specific volume compared to the control bread (Figure 5 and Supplementary Table S1). The most pronounced increases in weight (236.0 and 244.0 g) and specific volume (3.56 and 3.62 cm^3^/g) were detected in bread prepared by the addition of 15% HRF and HRHF, respectively.

Volume is a crucial qualitative factor since it affects how much air is captured and held in the finished product (77). Regarding volume, our results reported no significant differences in volume compared to the control except for the bread + HRF 5%, which was significantly lower than the control. Rashid and Dutta (22) reported that using rice husk-derived carboxymethyl cellulose improved both batter viscosity and cupcake volume.

The primary criterion for describing the quality of bread is its specific volume. It is best to choose bread with a higher specific volume. In our case, the reduction in wheat proteins (gluten) caused by the addition of rice bran flour to wheat flour is blamed for the decrease in the specific loaf volume of bread, which reduces air entrapment in the dough (64). Previous studies have linked the dilution of the gluten network, which in turn affects gas retention rather than gas production, to the negative effects of fiber in rice bran supplementation on dough structure (64). Bran supplements typically degrade the structure, resulting in decreased bread volume and crumb elasticity. Also, Wunthunyarat et al. (78), reported a specific volume that varies between 1.49 and 1.75 cm^3^/g in bread prepared with brown rice flour.

#### 3.3.5. Color

**Figure 6:**
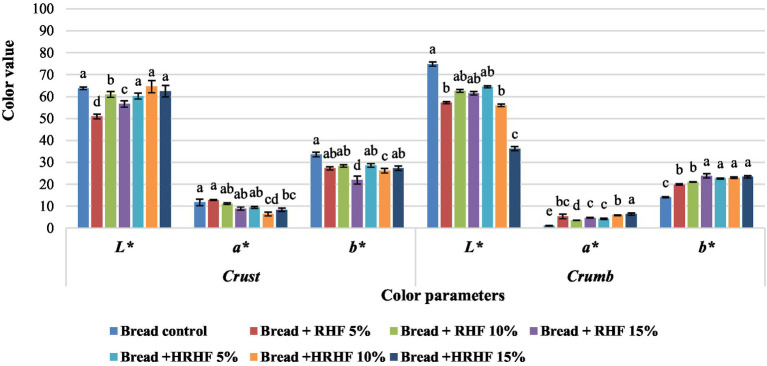
Color values of raw materials (*a*) and bread samples (*b*). Each data is the average ± standard deviation across three distinct replications. According to Duncan’s test for bread samples, the letters display significant differences at *p* ≤ 0.05.

**Figure 7:**
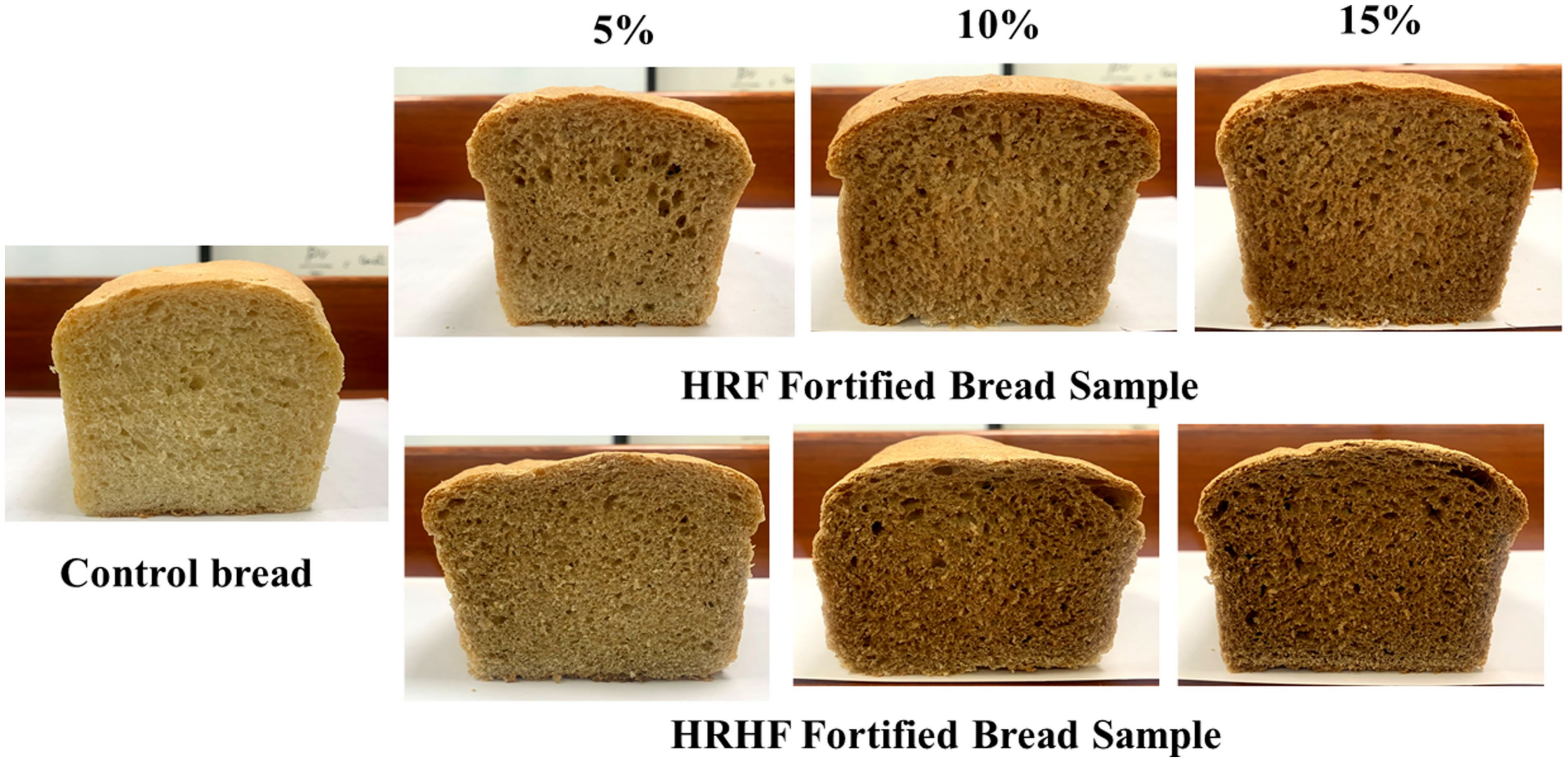
Pan bread loafs substituted with different levels of HRF and HRHF.

Color is a very important criterion for the initial acceptability of baked products by the consumer. The color parameter values of bread with several HRF and HRHF additions are shown in Figures 6, 7. Generally, the results indicated no constant trending in crust color values (lightness, yellowness, and redness) in response to increasing the fortification level of HRF and HRHF. On the other hand, the data showed a general decrease in lightness (L^*^) in fortified bread samples, while both the yellowness (b^*^) and redness (a^*^) have increased in fortified bread samples compared to the control. In this context, of Tuncel et al. (73) and Sallam et al. (27) reported that the inclusion of rice bran in wheat bread increased the redness and yellowness of the final product.

These changes might be attributed to the increase in pigmented constitutes, especially anthocyanins, due to fortification with both HRF and HRHF. These color alterations, often known as “color-deepening,” happen in red rice due to the oxidation of proanthocyanidin, which creates intramolecular linkages (79). Further, other brownish pigmented compounds like melanoidins and other colors are produced by the oxidation or degradation of phenolic compounds or due to the occurrence of Maillard reaction during baking (74, 80).

#### 3.3.6. Sensory analysis

**Figure 8:**
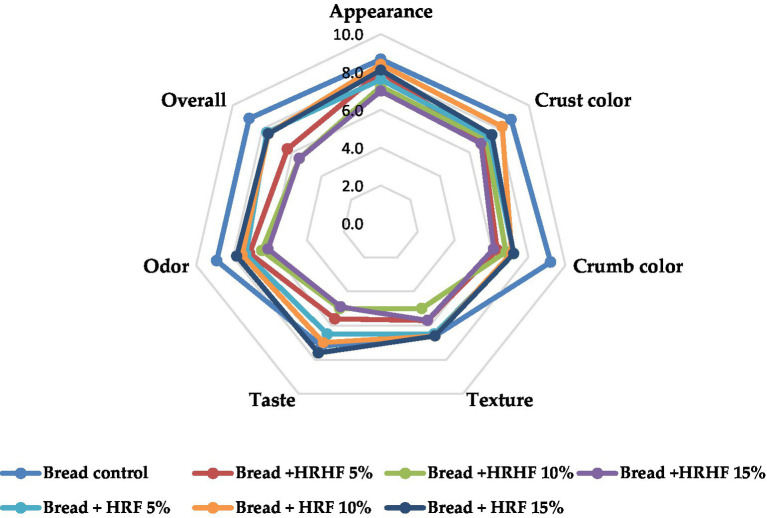
Radar chart showing the sensory rating of pan bread samples.

According to the sensory evaluation of samples, the addition of HRF and HRHF at all levels has changed the sensory qualities of supplemented bread when compared to the control sample (Supplementary Table S2 and Figure 8). These changes are primarily related to the appearance, crust and crumb colors, and odor attributes. According to the ANOVA test, all supplemented bread products are significantly different from the control sample. Whereas only two samples (bread + HRHF 10% and 15%) were liked differently from the control. On the other hand, changes in texture were not relevant. Due to that, the combined effects of these attributes have to the production of three bread samples with no significant differences from the control (bread + HRF of 5%, 10%, and 15%) as per the overall ratings. Further, all bread samples supplemented with HRHF were significantly different from the control.

These results are consistent with those of Sallam et al. (27), who found that composite bread with different amounts of defatted rice bran significantly altered the sensory properties compared to control bread. It was noted that the colors of the crust and crumb seemed to be a crucial factor in determining whether a consumer would initially accept the bread. The dark color was not appealing at increasing substitution levels, as seen by the lowest crust and crumb color scores for pan bread made with defatted rice bran at the highest substitution level (20%) (27). Though consumer understanding of more healthy choices regarding bakery products like fiber-rich bread has started to increase, especially among the highly educated population, to reach out to a border population from different educational levels, studies indicated that better placement on supermarket shelves as well as improving communication with consumers by intruding a clearer and more concise message would improve the sales of such products.

#### 3.3.7. Overall evaluation of bread samples

**Figure 9:**
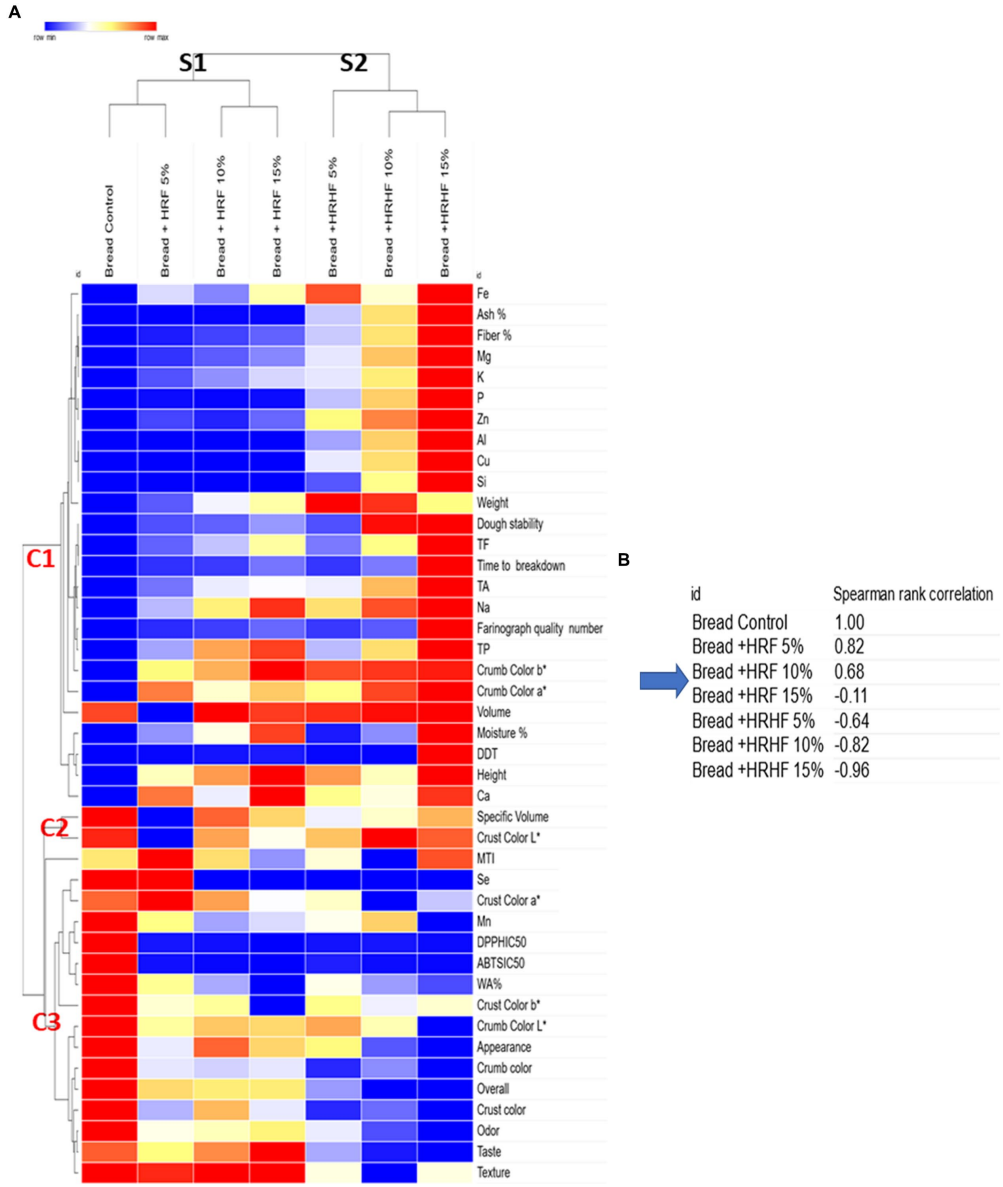
Hierarchical clustering of samples and their variables based on a heatmap generated using the *z*-score values **(A)** followed by nearest neighbors Spearman rank correlation test showing the closest sample to the control **(B)**.

Further, the chemo-physical, rheological, and sensor properties of the control and supplemented pan bread were assessed using the same method as the raw materials. In Figure 9A, variables are shown in a gradient color in correspondence to the mean value (blue below the mean and red above the mean). Based on sample type, bakery products were clustered into two groups, separating the control and RHF-supplemented products (S1) from the HRHF-substituted products (S2). This is in line with the results of the overall acceptability of samples, where both the control and bread substituted with RHF at all levels have no significant differences. Further, the variables clustered into two groups (C1 and C3) and one minor group (C2). Cluster C1 was responsible for separating the sample bread + HRHF 15% from the rest due to the overrepresentation of two macro components (fiber % and ash %), seven elements (Mg, P, Al, Cu, Si, Zn, K), three rheological parameters (DDT, farinograph quality number, and time to break down), and TA content. On the other hand, control bread sample was separated by C3 from the rest of the samples due to the high ratings of three sensory attributes (appearance and crust and crumb colors), two color parameters (crumb color L^*^ and crust color b^*^), one rheological parameter (WA %), while having the lowest antioxidant activity against both ABST and DPPH due to having the lowest concentrations of TP, TA, and TF.

Additional to the heatmap, a similarity matrix was generated from the clustered values, and then samples were ranked based on Spearman correlation to identify the nearest neighbor to the control sample (Figure 9B). Thereby, it was revealed that bread with HRF 5% and 10% additions was the closest to the control, followed by HRF at 15%. HRHF-fortified samples, on the other hand, deviated from the control proportionally to their addition level.

**Figure 10:**
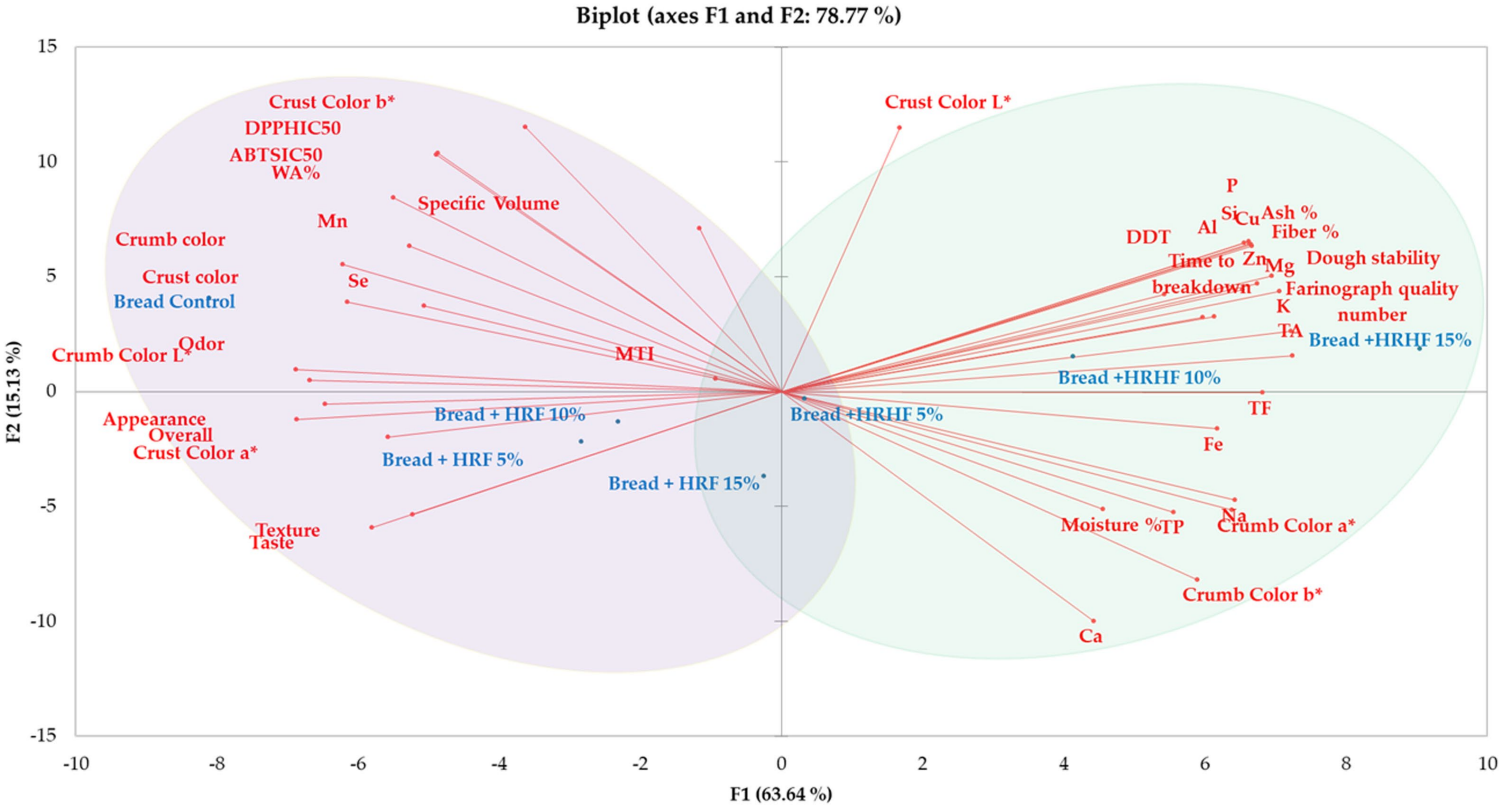
Principal component analysis of samples’ variables. Variables are colored in blue and observations in red.

We further performed principal component analysis to gain additional information regarding the correlation between variables in relation to their samples. According to the PCA-bi plot, two principal components could explain 78% of the variation between samples (Figure 10). Principal component one (F1) has separated samples successfully based on their type of substitution and their similarity to the control (HRF 5%, 10%, and 15% along with bread control in $-F_{1}$ and HRHF 5%, 10%, and 15% in $+F_{1})$ .

Two regions were identified by PCA. The first region (marked in light violet) identified the fortified samples that were correlated with desirable color and sensory qualities (HRF 5% and 10%). The second region (marked in light green) contained the fortified samples that were proved to have higher contents of elements, natural products, and macro components (ash, moisture, and fiber; *p* ≤ 0.05). In this context, previous reports indicated positive correlations between both fiber content and water holding capacity and ash content and the total elemental concentration in various food matrices (19, 45, 48).

Further, they possessed higher antioxidant activity, farinograph quality parameters, and physical quality parameters. A third minor region intersecting the two main regions contained the HRF addition at 15% and the HRHF at 5%. These samples possessed moderate values for the most important parameters (sensory qualities, levels of natural products, etc.).

Considering that the sensory parameters of bread + HRF 15% were not significantly different from the control bread (except for crust and crumb colors), we presume that the deviation between them was mainly caused by the higher content of macro components, natural products, and elements, in addition to the physical and farinograph parameters. Thus, we conclude that the closest samples to retain beneficial nutritional values and sensorial qualities closest to the control bread among the fortified samples are bread + HRF 5% and 10%.

## 4. Conclusion

Hassawi rice husk is a waste product of the rice milling industry, and its fortification in food is an economical way to enrich the nutritional value of various processed foods. This study demonstrated that Hassawi rice flour and husk have high nutritional value and can be used as nutrient-rich sources in the preparation of bread. It is possible to formulate high-fiber bread by incorporating HRF and HRHF up to 15% while retaining ratings above 5 “the neutral mark” on the sensory scale. However, samples fortified with HRF presented better sensory qualities. On the other hand, fortification with HRHF provided the highest concentrations of biological active molecules. In both cases, dough development and stability time increased, while water absorption, volume, and specific volume decreased. Based on the principal component analysis, the sample fortified with HRF at 5% and 10% were the closest to retaining acceptable sensory and rheological qualities while having a higher health benefit. To conclude, the current study has confirmed the feasibility of supplementing wheat flour with HRF and HRHF in the preparation of bread, which helps in the reduction of food waste that causes environmental issues and pollution. In the future, we recommend investigating the effects of incorporating flatbreads, such as Balady bread, with HRF and HRHF due to their lower flour quality requirements, which allows for the incorporation of additional ingredients into flatbreads without compromising texture when compared to pan breads.

## Data availability statement

The original contributions presented in the study are included in the article/Supplementary material, further inquiries can be directed to the corresponding authors.

## Ethics statement

The studies involving humans and human participants were reviewed and approved by the ethical guidelines of King Faisal University (approval number KFU-REC-2022-NOV-ETHICS819). The studies were conducted in accordance with the local legislation and institutional requirements. The participants provided their written informed consent to participate in this study.

## Author contributions

HE-B, HE, AA, HM, HA-O, KR, and MM: conceptualization, methodology, validation, writing—review and editing, and visualization. HE-B, AA, HM, and MM: software. HE-B, HE, AA, KR, and MM: formal analysis. HE-B, AA, and KR: investigation and supervision. HE, HM, and HA-O: resources. HE-B, KR, and MM: data curation. HE-B, MM, and HM: writing—original draft preparation. HE-B and HM: project administration. HE-B, HE, AA, HA-O, and KR: funding acquisition. All authors contributed to the article and approved the submitted version.

## Funding

This research work was supported and funded by the Deputyship for Research and Innovation, Ministry of Education in Saudi Arabia (Project number INSTR001).

## Conflict of interest

The authors declare that the research was conducted in the absence of any commercial or financial relationships that could be construed as a potential conflict of interest.

## Publisher’s note

All claims expressed in this article are solely those of the authors and do not necessarily represent those of their affiliated organizations, or those of the publisher, the editors and the reviewers. Any product that may be evaluated in this article, or claim that may be made by its manufacturer, is not guaranteed or endorsed by the publisher.

## Supplementary material

The Supplementary material for this article can be found online at: https://www.frontiersin.org/articles/10.3389/fnut.2023.1240527/full#supplementary-material

## Footnotes

^1^ https://software.broadinstitute.org/morpheus/
