# Supplementary material for: The potential use of Indian rice flour or husk in fortification of pan bread: assessing bread’s quality using sensory, physicochemical, and chemometric methods
Source: Front Nutr. 2023 Sep 15;10:1240527. doi: 10.3389/fnut.2023.1240527 (PMC10540694; doi:10.3389/fnut.2023.1240527)
Supplement: Supplementary file 1 [file Data_Sheet_1.docx]

Supplementary Material

The potential use of Indian rice flour or husk in fortification of pan bread: Assessing bread's quality using sensory, physicochemical, and chemometric methods

**Haiam O. Elkatry, Hossam S. El-Beltagi, Abdelrahman R. Ahmed, Heba I. Mohamed, Hala Hazam Al-Otaibi, Khaled M. A. Ramadan , Mohamed A. A. Mahmoud**

# Supplementary Figures and Tables

## Supplementary Figures


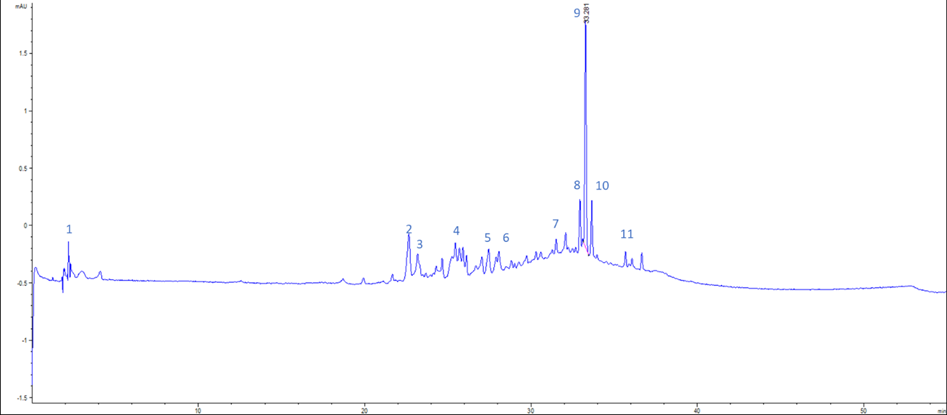


**Supplementary Figure 1.** Chromatogram of total phenolic compounds in HRF extract.


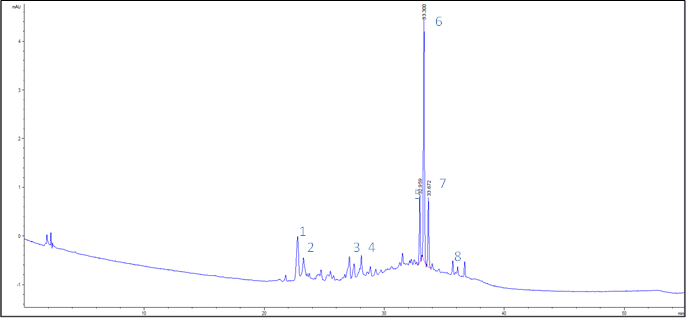


**Supplementary Figure 2.** Chromatogram of total phenolic compounds in HRHF extract.

**Supplementary Figure 3.** Chromatogram of tocopherols, tocotrienols, and γ-oryzanol compounds content in extracts of HRF (A) and HRHF B.

**Control Bread**

**Bread +HRF 5%**

**Bread +HRF 10%**

**Bread +HRF 15%**

**Bread +HRHF 5%**

**Bread +HRHF 15%**

**Bread +HRHF 10%**

**Supplementary Figure 4.** Farinogram of bread substituted with different levels of HRF and HRHF flour.

## Supplementary Tables

**Supplementary Table S1** Weight, height, volume, and specific volume of bread samples.

| **Specific volume**  **(cm^3^/g)** | **Volume**  **(cm^3^)** | **Height**  **(cm)** | | | **Weight**  **( g )** | | **Substitution**  **(%)** | |
| --- | --- | --- | --- | --- | --- | --- | --- | --- |
| 3.62 ± 0.03^a^ | 821.67 ± 7.64^a^ | 8.00±1.00^a^ | | | 227.00 ± 1.00^e^ | | **Control** | |
| 3.24 ± 0.04^d^ | 743.33 ± 10.41^b^ | | 7.33 ± 0.58^a^ | 229.33 ± 0.58^d^ | | **5** | | **Bread +HRF %** |
| 3.56 ± 0.02^a^ | 831.67 ± 7.64^a^ | | 7.67 ± 0.58^a^ | 233.33 ± 0.58^c^ | | **10** | |  |
| 3.49 ± 0.04^b^ | 823.33 ± 5.77^a^ | | 7.67 ± 0.58^a^ | 236.00 ± 1.00^b^ | | **15** | |  |
| 3.38 ± 0.02^c^ | 825.00 ± 5.00^a^ | | 8.00 ± 1.00^a^ | 244.00 ± 1.00^a^ | | **5** | | **Bread +HRHF %** |
| 3.42 ± 0.02^c^ | 830.00 ± 5.00^a^ | | 7.33 ± 0.58^a^ | 242.67 ± 1.53^a^ | | **10** | |  |
| 3.51 ± 0.02^b^ | 831.67 ± 2.89^a^ | | 6.67±0.58 ^a^ | 237.00 ± 1.00^b^ | | **15** | |  |

1. Each data is the average ± standard deviation across three distinct replications. According to Duncan's test, letters in the same column display a significant difference at *p* ≤ 0.05.

**Supplementary Table S2.** Sensory evaluation of bread samples.

| **Sample** | | **Appearance** | **Crust color** | **Crumb color** | **Texture** | **Taste** | **Odor** | **Overall** |
| --- | --- | --- | --- | --- | --- | --- | --- | --- |
| **Bread control** | | 8.7±0.9^a^ | 8.8±0.4^a^ | 9.2±1.0^a^ | 6.6±2.8^a^ | 7.2±2.6^a^ | 8.9±0.8^a^ | 8.9±0.8^a^ |
| **Bread +HRF %** | **5** | 7.6±1.3^bcd^ | 7.3±0.9^bc^ | 7.2±2.1^b^ | 6.5±2.2^a^ | 6.5±1.9^ab^ | 7.3±1.3^bcd^ | 7.7±1.1^ab^ |
|  | **10** | 8.4±0.8^ab^ | 8.2±0.9^ab^ | 7.1±2.0^b^ | 6.6±2.1^a^ | 7.0±2.0^ab^ | 7.5±1.7^bc^ | 7.6±1.4^ab^ |
|  | **15** | 8.1±0.9^abc^ | 7.5±1.2^bc^ | 7.2±1.8^b^ | 6.6±2.1^a^ | 7.6±1.8^a^ | 7.8±1.4^ab^ | 7.6±1.3^ab^ |
| **Bread +HRHF %** | **5** | 8.0±1.0^abcd^ | 6.9±1.7^c^ | 6.3±2.1^b^ | 5.7±3.0^a^ | 5.6±2.4^ab^ | 7.1±0.9^bcd^ | 6.3±1.9^bc^ |
|  | **10** | 7.2±1.0^cd^ | 7.1±0.6^c^ | 6.8**±**1.6^b^ | 5.0±2.7^a^ | 5.0±2.1^b^ | 6.4±0.7^cd^ | 5.5±2.0^c^ |
|  | **15** | 7.0±0.7^d^ | 6.8±0.4^c^ | 6.1±0.8^b^ | 5.7±2.8^a^ | 4.9±2.0^b^ | 6.1±0.8^c^ | 5.5±2.0^c^ |

Each value represents the mean (±SD) of three different replications. The different letters on the same column show a significant difference according to Duncan’s test at *p* ≤ 0.05.
